# Therapeutics controversies in antineutrophilic cytoplasmic antibody-associated vasculitis

**DOI:** 10.1007/s10157-025-02693-w

**Published:** 2025-05-20

**Authors:** Ken W. T. Chau, Zaw Thet, Logan S. Gardner, Tien K. Khoo, Alfred K. Lam, Dwarakanathan Ranganathan

**Affiliations:** 1 School of Medicine and Dentistry, Griffith University, G40 8.34, Gold Coast, QLD 4222 Australia; 2 Rockhampton Hospital, Rockhampton, QLD Australia; 3 Alfred Hospital, Melbourne, VIC Australia; 4 Graduate School of Medicine, University of Wollongong, Wollongong, NSW Australia; 5 Gold Coast University Hospital, Gold Coast, QLD Australia; 6 Mayne Medical School, University of Queensland, Brisbane, QLD Australia; 7 Kidney Health Service, Royal Brisbane and Women’s Hospital, Brisbane, QLD Australia

**Keywords:** ANCA, Vasculitis, Therapies, Plasma exchange

## Abstract

Anti-neutrophil cytoplasmic antibody (ANCA)-associated vasculitis (AAV) is characterized by inflammation of small- to medium-sized blood vessels with ANCAs. Induction therapy has historically involved cyclophosphamide (CYC) and glucocorticoids (GCS), with rituximab (RTX) emerging as a preferred alternative. Studies addressed the efficacy of RTX in severe kidney diseases, showing results comparable to CYC. In severe diseases that are unsuitable for high-dose CYC, combined RTX and low-dose CYC demonstrate promising results. There is a recent trend to administer low-dose GCS to reduce infections and other complications. Recent trials have questioned the broader role of plasma exchange (PLEX), influencing guidelines’ updates. Current indications for PLEX include severe kidney dysfunction, positive glomerular basement membrane antibody, and diffuse alveolar hemorrhage that requires oxygen support at presentation. Maintenance therapy options include azathioprine, RTX, and methotrexate. RTX's efficacy as a maintenance agent was demonstrated in trials. However, controversies in dosing frequency persist. The optimal duration of maintenance therapy remains debated, with guidelines suggesting 18–48 months. More recent studies proposed longer durations to reduce the risk of relapse, thus challenging existing guidelines. Complement involvement in AAV has been increasingly recognized. Studies show that complement 3 depositions in the glomeruli correlate with severe disease and complement inhibitors such as avacopan demonstrated efficacy in the management of AAV. Other complement inhibitors, such as eculizumab and vilobelimab, are being explored. In conclusion, AAV management has made significant advances, but controversies persist. Future research should refine therapeutic regimens and explore novel targeted treatments for personalized medicine in AAV.

## Introduction

Anti-neutrophil cytoplasmic antibody (ANCA)-associated vasculitis (AAV) encompasses a group of complex autoimmune diseases characterized by inflammation of small- to medium-sized blood vessels and the presence of ANCA, with a prevalence of 46–184/million people [1]. Historically, AAV has been divided into distinct entities, such as granulomatosis with polyangiitis (GPA), microscopic polyangiitis (MPA) and eosinophilic granulomatosis with polyangiitis (EGPA), each with its unique clinical presentation and organ involvement. The pathogenic role of ANCA, especially against proteinase-3 (PR3) and myeloperoxidase (MPO), was identified in 1988 and 1990 respectively [2–4], has been central to our understanding of disease mechanisms, leading to its naming and the development of diagnostic strategies.

Despite significant advances in therapeutic approaches, AAV remains a formidable challenge in medicine due to its potential for rapid progression, end-organ damage, and the complexities associated with long-term management. Although the last two decades have witnessed a transformative change in the prognosis of AAV with the advent of new immunosuppressive therapies, challenges remain in balancing the treatment's efficacy and its potential adverse effects.

This review aims to provide current and long-standing controversies surrounding treatment options, dosing, duration and recent advancements in novel therapeutic strategies of this complex multisystem disease.

## Induction therapy: glucocorticoids with cyclophosphamide or rituximab

Induction therapy has often involved the use of cyclophosphamide (CYC) and standard-dose glucocorticoids (GCS). Rituximab (RTX), a type I anti-CD20 monoclonal antibody, has been established as an alternative agent to avoid complications and side effects of CYC. RTX is equally effective in inducing remission when compared to CYC in two randomized controlled trials (RCT) [5, 6], which has led it to become the standard of care in Kidney Disease: Improving Global Outcomes (KDIGO) 2024 guidelines for the management of patients with AAV [7].

Still, there is uncertainty as to whether RTX is truly suitable in patients with severe kidney failure. The Rituximab versus Cyclophosphamide for ANCA-Associated Vasculitis (RAVE) trial [5] excluded patients with severe kidney disease (creatinine > 354 μmol/L [4 mg/dL]), while the RITUXVAS trial [6] included patients with severe kidney dysfunction and participants who had received two doses of IV CYC in addition to RTX (76% RTX vs 82% CYC, *p* = 0.68). As the RITUXVAS study included the use of CYC in the RTX group, it leaves the question open as to whether RTX is truly suitable in patients with severe kidney failure.

This question was addressed by two further studies. Geetha et al. conducted a retrospective study of 37 patients with an estimated glomerular filtration rate (eGFR) of < 20 ml/min/1.73 m^2^ treated with RTX and GCS with or without CYC [8]. RTX was dosed at 375 mg/m^2^ once a week for 4 weeks in 12 patients. In the CYC group, 7 patients received oral CYC and 18 received pulse IV CYC. The median follow-up was 973 days, and the median eGFR was 13 ml/min/1.73 m^2^ compared to the RAVE trial, which was 53.8 ± 29.8 ml/min/1.73 m^2^. There was no statistical difference between treatment groups for remission, renal recovery, and death.

Casal Moura et al. conducted a retrospective study of 251 patients with renal AAV and severe kidney disease (eGFR < 30 ml/min/1.73 m^2^), with or without plasma exchange (PLEX) [9]. 161 received CYC (oral 2 mg/kg for 6 months), and 64 received RTX (IV 375 mg/1.73 m^2^ weekly for 4 doses). Both arms received tapering GCS that were not part of a pre-established protocol. This study concluded that RTX was comparable to CYC in remission induction at 6 months, and the addition of PLEX did not show a statistically significant benefit.

Recent data from the French Vasculitis Study Group Registry had shown that RTX achieved a higher rate of remission compared to intravenous CYC in GPA, particularly in PR3-ANCA disease (73.1% RTX versus 40.1% CYC, RR = 1.82 [95% CI, 1.22–2.73]). However, it did exclude patients with severe kidney disease (creatinine > 354 μmol/L [4 mg/dL]) and is limited by the nature of the retrospective registry data [10].

In patients presenting with severe AAV and pre-existing comorbidities, the use of high-dose CYC is dissuaded. Retrospective studies have shown promising results with combined RTX, low-dose CYC and GCS induction therapy [11, 12]. When comparing combined treatment against a historical cohort with only CYC and GCS, Gulati et al. observed comparable rates of AAV remission, higher rates of recovery from dialysis-dependent kidney failure, and improved end-stage kidney disease (ESKD)-free survival [11]. The addition of CYC to RTX may act to suppress a broader range of immune effectors, such as neutrophils, macrophages, and T cells, providing more rapid control of inflammation and improved recovery.

In selected patients with AAV where RTX is contraindicated due to severe adverse effects or patients with refractory disease and there is a strong preference to avoid CYC, alternative agents are needed. Mycophenolate has been shown to have a remission rate like that of CYC for non-life-threatening AAV. However, it is associated with a higher risk of relapse in those with PR3-ANCA disease, thereby limiting its indication toward mild–moderate MPO-ANCA disease [13]. Obinutuzumab is a type II anti-CD20 monoclonal antibody that can induce a deeper and more durable B cell depletion compared to RTX [14]. In a recent phase 2 study (NOBILITY trial) of obinutuzumab in the treatment of lupus nephritis [15], there are early promising results and a reassuring safety profile. Following this, a recent case series [16] included three patients with AAV who were treated with obinutuzumab due to a prior history of anaphylactic reaction to RTX. They concluded that treatment was well-tolerated in all patients and effective in inducing remission with respective success in inducing B cell depletion and a fall in ANCA titer.

Historically, many patients with AAV received long-term, high cumulative doses of GCS as part of their induction regimen. Adverse events related to GCS are well-described. Recently, there has been a trend toward minimizing exposure to high-dose GCS, especially in patients who are high-risk or frail [17–21]. The PEXIVAS [17] study demonstrated that a reduced-dose GCS regimen, compared to a more traditional, higher-dose regimen, provided a similar benefit for the composite outcome of ESKD or death and was associated with a decreased risk of infection. The reduced-dose regimen started with daily pulsed methylprednisolone (1–3 doses for a maximum total dose of 3 g) and 1 week of high-dose oral GCS. There are concerns that pulsed intravenous methylprednisolone may increase the risk of infection, but clinical trial data are lacking.

## Role of plasma exchange in induction therapy in AAV

Controlled trials have not consistently demonstrated a benefit in the use of PLEX in patients with AAV apart from several specific indications. The core theory behind the process is the rapid removal of a pathological autoantibody to reduce inflammation and organ injury.

The first indication for PLEX is in patients with rapidly deteriorating or severely impaired kidney function. This benefit was initially demonstrated in an RCT of patients with predominantly AAV [22]. Results demonstrated that patients presenting with severe kidney dysfunction (creatinine > 500 μmol/L [5.66 mg/dL] or on dialysis) had a greater probability of renal recovery, odd ratios (OR) = 2.42 (*p* = 0.041). This was followed by the Methylprednisolone versus Plasma Exchange (MEPEX) trial in 2007 [23], which studied patients (*n* = 137) with severe renal dysfunction (creatinine > 500 μmol/L). The patients were randomized to either 7 cycles of PLEX or 3 pulses of 1 g methylprednisolone (MP) in addition to CYC and oral GCS. The trial showed that PLEX is superior to MP. The use of PLEX was associated with a significant improvement in renal recovery at 3 months (69% PLEX group vs 49% MP group, *p* = 0.02) and a 24% risk reduction of ESKD at 12 months compared to MP. Short-term benefits of PLEX were not maintained at a median of 3.95 years of follow-up*.* Furthermore, there was a non-significant increase in infection-related deaths in participants who received PLEX [23]. For these reasons, the PEXIVAS trial was conducted.

The PEXIVAS trial involved 704 patients with an eGFR < 50 ml/min/1.73 m^2^ [17]. Patients were randomized to either PLEX or no PLEX in addition to GCS and either RTX or CYC. Furthermore, patients were randomized to either low- or standard-dose GCS. The trial failed to demonstrate a benefit in the composite endpoint of death or ESKD, with a hazard ratio of 0.86 (95% CI 0.65–1.13; *p* = 0.27) when compared to standard induction therapy alone. It should be noted that baseline kidney biopsy was not mandatory in study inclusion, and therefore, patients with chronic sclerosis might have been included. Additionally, study inclusion criteria were broad, and the study may have been underpowered to evaluate patients with crescentic disease, serum creatinine > 500 μmol/L (5.6 mg/dl) or those who required dialysis.

A recent meta-analysis that included PEXIVAS and eight other RCTs assessed the effect of PLEX in patients (*n* = 1060) with AAV [24]. PLEX reduced the risk of ESKD at 12 months (RR 0.62 95% CI 0.39–0.98), but there were no effects on mortality (RR 0.90 95% CI 0.64–1.27). However, there was an increased risk of infection at 12 months in the PLEX group (RR 1.27 95% CI 1.08–1.49). Based on this data, PLEX in patients with a low risk of progression to ESKD is likely not beneficial. Sub-analysis of this data had shown that PLEX could be beneficial in patients with serum creatinine > 300 μmol/L (3.4 mg/dL). Therefore, the European Alliance of Associations for Rheumatology (EULAR) and the KDIGO 2024 guidelines recommend PLEX in patients with serum creatinine > 300 μmol/L (3.4 mg/dL) [7, 25].

Another indication of PLEX is in AAV patients with concurrent positive anti-glomerular basement membrane (GBM) antibodies. Double-positive disease is uncommon although not as rare as expected given the individual prevalence of the two conditions [26]. A study reported that 50% of anti-GBM cases and 10% of patients with AAV are double-positive, with an associated worse prognosis than the diseases individually [27]. A review of the literature of published cases by Uto et al. demonstrated a survival rate of those who received PLEX of 74% compared to 53% of those who received immunosuppression alone [26].

The final indication for PLEX is diffuse alveolar hemorrhage (DAH), which is defined by the presence of hemoptysis, multi-lobar infiltrates, hypoxia, and progressive anemia. While there is currently no dedicated randomized trial to guide therapy, a retrospective study by Klemmer et al. demonstrated resolution in all 20 patients with DAH treated with PLEX [28]. All patients in the study had AAV and were treated with induction methylprednisolone and/or intravenous CYC with PLEX performed daily until clinical improvement, which was defined as improvement in oxygenation and stabilization or improvement in pulmonary infiltrates and hematocrit. Subsequently, exchanges were performed every two days until resolution. The PEXIVAS study also included a subgroup of 191 patients with DAH, and this resulted in what is currently the only RCT in this subpopulation. Due to the heterogeneity of the DAH cohort, they were unable to demonstrate a statistically significant treatment benefit of PLEX in this group, although there was a slight trend to favor PLEX in patients with severe DAH with a hazard ratio of 0.64 (95% CI 0.33–1.24) [17].

## Maintenance therapy for AAV

After induction treatment in AAV, maintenance therapy is required to prevent relapse. Azathioprine, methotrexate, mycophenolate, and RTX have been studied in this treatment stage. In an RCT of 159 patients with 12 months of maintenance therapy, methotrexate was found to be equally effective in relapse prevention when compared to azathioprine (33% versus 36%, *p* = 0.71). There were, however, more severe adverse events with methotrexate leading to discontinuation of treatment (HR = 1.65, CI 0.65–4.18) [29]. Mycophenolate, on the other hand, when used as maintenance, was less effective in relapse prevention compared to azathioprine but had no difference in infection rate [30, 31].

Until recently, azathioprine had been the standard immunosuppressive used for the maintenance of remission in AAV. Current evidence of RTX use in maintenance therapy has demonstrated that RTX is the preferred maintenance agent in AAV. In the Maintenance of Remission Using Rituximab in Systemic ANCA-associated Vasculitis (MAINRITSAN) trial, the use of RTX as a maintenance agent after CYC induction had decreased major but not minor relapses compared to azathioprine [32]. The efficacy of RTX as a maintenance agent was confirmed again in the Rituximab versus Azathioprine as Therapy for Maintenance of Remission for Anti-Neutrophil Cytoplasm Antibody-associated Vasculitis (RITAZAREM) trial which found 1000 mg of RTX every 4 months resulted in decreased major and minor relapses compared to 2 mg/kg/day azathioprine, without significant difference in adverse effects such as infection [33]. Still, it is debatable whether to use RTX in all patients as there is a concern of hypogammaglobulinemia that may require long-term immunoglobulin therapy.

The optimal dosing regimen and frequency of RTX in the maintenance of AAV remains controversial. The MAINRITSAN-2 trial compared the efficacy of a fixed dose regimen with a bi-annual 500 mg RTX or a tailored infusion dose of 500 mg based upon the reappearance of CD19 + B cells and/or ANCA titers [34]. Both regimens prevented relapse with a lower relapse rate of 9.9% in the fixed regimen group versus 17.3% in the tailored infusion group (*p* = 0.22), though there was a trend to favor a fixed dose regimen. The tailored infusion group was observed to require fewer doses (median 3 versus 5 infusions). Serious adverse events were comparable between the fixed regimen and tailored infusion group: 38.3% versus 32.1% (*p* = 0.51).

With regard to RTX’s optimal dosing frequency in fixed regimen studies, the RITAZAREM trial dosed 1000 mg every 4 months [33], whereas the MAINRITSAN trial dosed 500 mg every 6 months [32, 34]. While both studies demonstrated the superior efficacy of RTX compared to azathioprine and a comparable safety profile, the optimal dose and the dosing frequency remain unclear. Belimumab, a human monoclonal antibody that inhibits B cell activating factor, has recently been tested in AAV as an additional agent to azathioprine and GCS as maintenance therapy, and there was no benefit compared to placebo [35].

## Optimal duration of maintenance therapy

The duration of maintenance treatment was evaluated in a randomized prospective study across 33 centers and 11 countries involving 117 patients [36]. This study included patients diagnosed with AAV and had either renal or other organ-threatening diseases. Patients were recruited 18–24 months post-diagnosis and in remission. Groups were randomized to withdraw azathioprine at 24 months from diagnosis or continue until 48 months. Relapse occurred in 63% of the withdrawal group compared to 22% in those continuing therapy. Major relapses occurred in 35% of the withdrawal group compared to 14% of the ongoing therapy group. Progression to ESKD occurred in 4 patients (7.8%) in the withdrawal group versus none in the continuing therapy group (*p* = 0.012). Severe adverse events occurred in 3(6%) patients in the withdrawal group versus 9(15%) patients in the group continuing therapy (*p* = 0.13). Mean eGFR at the end of follow-up in the ongoing therapy group increased by 2.5 ml/min/1.73 m^2^ compared to a reduction of 3.3 ml/min/1.73 m^2^ in the withdrawal group (*p* = 0.01).

The MAINRITSAN-3 trial, an RCT across 39 centers in France in 2020 [37], enrolled 97 patients who had completed 18 months of RTX maintenance without any major relapses and were in complete remission. This study evaluated the efficacy of extended RTX maintenance therapy at 500 mg every 6 months for a further 18 months as compared to placebo. The primary endpoint of relapse-free survival at 46 months was achieved in 96% of patients in the treatment arm compared to 74% in the placebo arm (RR = 0.16, *p* = 0.008). Serious adverse events were comparable between RTX (24%) and placebo (30%). This study concluded that extended maintenance RTX treatment to 36 months is associated with a significantly lower risk of relapse compared to standard 18 months of maintenance therapy.

While the above studies suggest that a longer duration of therapy beyond 24 months was beneficial, recently, a pooled analysis of 277 patients from the MAINRITSAN trials reported that extending RTX to 36 months did not improve relapse-free survival at month 84. Of note, there was still a trend to favor fixed dosing interval extended RTX to 36 months (adjusted HR for overall relapse 0.69 [95% CI 0.38–1.25]), and this did not increase the rate of serious infections [38].

Guidelines recommend varied treatment durations dependent on individual circumstances (Table 1) [7, 25, 39]. The KDIGO 2024 guidelines suggest the optimal duration for remission maintenance with either RTX or azathioprine, and low-dose GCS is 18–48 months [7]. The EULAR 2022 guidelines recommended that maintenance therapy should be continued for at least 24 months, a longer duration of up to 48 months should be considered in relapsing patients or those with an increased risk of relapse, taking into account the risk of continuing immunosuppression and patient preference [25]. The American College of Rheumatology/Vasculitis Foundation (ACR/VF) group in 2021 recommended that the duration of treatment should be guided by the patient’s clinical condition, preferences, and values [39].

**Table 1:** Recent studies/guidelines on AAV maintenance therapy duration

Landmark studies/society guidelines	Cohort size	Key results	Conclusion/recommendation
Karras 2017 [36]	117	22% relapse in the continuing azathioprine group vs 63% in the withdrawal group 24–48 months after AAV diagnosis	Continuing azathioprine maintenance for 48 months results in fewer relapses, and improved eGFR at the end of follow-up without an increase in severe adverse events
MAINRITSAN-3 2020 [37]	97	4% relapse in the continuing RTX group vs 26% in placebo 18–36 months after AAV diagnosis	Extending maintenance RTX to 36 months is associated with a lower risk of relapse and comparable adverse events observed at 46 months
MAINRITSAN pooled analysis [38]	277	84-month major relapse-free survival was 49% for AZA, 70% for 18-month fixed RTX, 49% for 18-month-tailed RTX, 74% for 36-month tailored/fixed RTX, and 83% for 36-month fixed/fixed RTX	Extended fixed dose maintenance RTX from 18 to 36 months did not achieve a statistically significant difference in relapse-free survival at 84 months. However, there was a trend favoring extended maintenance while both had comparable adverse events
ACR/VF 2021 [39]	N/A	N/A	The duration of treatment should be guided by the patient’s clinical condition, preferences and values
EULAR 2022 [25]	N/A	N/A	Maintenance therapy should be continued for at least 24 months, up to 48 months in relapsing patients or those at increased risk of relapse
KDIGO 2024 [7]	N/A	N/A	Maintenance is recommended with either RTX or azathioprine, and low-dose GCS for 18–48 months. Treatment duration is dependent on individual circumstances

## Role of complement in ANCA vasculitis—novel therapeutic target

Kidney involvement in AAV is characterized by a paucity of immuno-complex deposition in the glomeruli. Consequently, complement consumption is not classically a component of the disease, and the presence of low complements often suggests an alternative pathology such as lupus nephritis.

The potential role of complement in AAV was elucidated in a study that demonstrated that 58% (68/126) of patients with crescentic AAV had glomerular immune complex deposits seen on electron microscopy, of which 87% had positive findings on immunofluorescence [40].

Chen et al. subsequently demonstrated C3 deposition in the glomeruli of 33% of patients [41]. Of note, those with C3 deposition had higher urinary protein 1.8 g/24 h versus 0.93 g/24 h (*p* < 0.01), higher initial serum creatinine 491.2 μmol/L (5.56 mg/dL) versus 354.8 μmol/L (4.01 mg/dL) (*p* < 0.01) and higher need for kidney replacement therapy at presentation 48.6% versus 28.0% (*p* < 0.05). The results suggest that complement deposition is common in AAV and associated with more severe kidney disease.

Another study explored the effect of serum C3 on prognosis in patients with AAV [42]. This retrospective study assessed 45 patients with AAV and divided the patients into those with high-normal C3 and low-normal C3 levels at diagnosis before treatment. All patients who had initial C3 results that were within the normal range were investigated by kidney biopsy and followed up for a mean of 55 months. Patients in the low–normal C3 group had higher creatinine at diagnosis, poorer long-term survival, and poorer ESKD-free survival. In contrast, patients with elevated C3 levels and crescentic or mixed histology, according to Berden's classification, had a significantly better prognosis at 6 years, 100% versus 40.7% (*p* = 0.046). Overall, it was identified that patients in the low C3 group had an increased risk of ESKD, OR = 7.1 (*p* = 0.03) though after adjustment for creatinine at diagnosis, statistical significance was not met.

The potential importance of the alternative pathway in AAV was demonstrated in a mouse model with selective defects in the classical, alternative and lectin-binding pathways in 2009 [43]. This study demonstrated that mice were protected from disease by impairing the alternative pathway by affecting C5 and Factor B. Five years later, the same investigators discovered that MPO-ANCA-induced necrotising crescentic glomerulonephritis in humanized mice was mediated by neutrophil C5a receptor/CD88 [44]. They reported that C5aR/CD88 engagement enhances inflammation, and C5a-like receptor engagement suppresses inflammation and the use of avacopan (antagonist of human C5aR/CD88) can ameliorate the process of AAV in mice subjects.

Avacopan is a potent selective inhibitor of the C5a receptor, and it has been studied in phase II [45, 46] and phase III RCT [18] in the treatment of AAV. The Avacopan Development in Vasculitis to Obtain Corticosteroid Elimination and Therapeutic Efficacy (ADVOCATE) trial tested the hypothesis that avacopan is effective in patients with AAV as a GCS-sparing agent when used in conjunction with RTX/CYC. Although GCS were still used in 75% of the avacopan group, the mean daily GCS dose was one-third of that of the GCS group (4 mg versus 12 mg). In terms of remission, avacopan was found to be of similar efficacy in achieving remission compared to tapered GCS at week 26 but was superior at maintaining remission at week 52 [18]. Serious adverse events were 33% higher in the GCS group. There were more deaths, life-threatening or serious adverse events and infections when compared to the avacopan group. There was, however, more abnormal liver function in the avacopan group (5.4% versus 3.7%), all of which resolved after withdrawal from the trial drug and other potentially hepatotoxic drugs. Avacopan has also been shown to benefit kidney function and albuminuria, especially in patients with eGFR < 20 ml/min/1.73 m^2^ [18]. The findings were consistent with previous studies in mice and humans [44–46].

Avacopan offers a more specifically targeted approach in the inflammatory pathway compared to traditional GCS. Although it is now recommended as an alternative to GCS in the KDIGO 2024 guidelines [7], its ability to serve as a true GCS-sparing agent remains controversial as most patients received GCS in the avacopan group. More studies are needed to determine its independent efficacy without GCS. Once established, the utility of a GCS-sparing agent would be particularly useful in vulnerable patients who cannot tolerate conventional GCS doses, such as those with significant frailty, severe osteoporosis, poorly controlled diabetes, or underlying psychotic disorders. Either way, clinicians should taper GCS where possible to minimize systemic adverse events.

After data indicating that C5a receptor inhibition results in better AAV treatment response, other complement inhibitors were tested. Eculizumab, a recombinant humanized monoclonal antibody against C5, prevents the formation of the complement membrane attack complex and has demonstrated effectiveness in case reports [47–49]. In a recent phase II trial of vilobelimab, a chimeric IgG4 kappa monoclonal antibody that inhibits C5a, has shown modest efficacy as a GCS-sparing agent in the treatment of AAV [50], though the trial was not powered to be non-inferior to GCS. Over time, emerging evidence highlights the important role of complement activation in AAV pathogenesis, in contrast to the traditional understanding of the paucity immuno-complex deposition in the glomeruli.

## Conclusion

AAV remains a complex and heterogeneous group of disorders with significant morbidity and mortality. The therapeutic landscape has evolved over the years, with novel treatments, such as biologics and targeted complement therapies, showing promise in disease management and improved patient outcomes. However, controversies persist in the management of AAV. Although current evidence suggests that RTX is a non-inferior alternative to CYC for the induction of AAV, there is limited data to support the use of RTX in AAV with severe kidney disease. Combining RTX and low-dose CYC in patients with severe AAV is a novel strategy, but the evidence remains limited. The role of PLEX in AAV remains debatable. Current evidence does not support the routine use of PLEX in non-severe AAV diseases except in specific indications. Avacopan appears efficacious in reducing reliance on GCS though further studies are awaited to determine if avacopan can replace GCS completely.

Azathioprine and RTX remained the most studied maintenance agents. The optimal duration of maintenance therapy for AAV, however, remains unclear. Recent trials support longer treatment periods (up to 48 months) to decrease the risk of relapse [36, 37], but the optimal duration should be carefully weighed based on the risk of adverse events from treatment and individual ANCA status as patients with MPO- and PR3-AAV have significantly different risks of relapse and patient preference.

Management of AAV has advanced significantly over the past decades. Future research should focus on refining therapeutic regimens to minimize the risk of adverse effects and explore novel treatments that target specific pathways implicated in the pathogenesis of the disease, which could usher in an era of personalized medicine in AAV.

## Data Availability

As this is a review article, no new data were generated or analyzed; therefore, all data supporting the findings presented here are available in the cited primary literature.

## References

[1] Watts RA, Mahr A, Mohammad AJ, Gatenby P, Basu N, Flores-Suarez LF. Classification, epidemiology and clinical subgrouping of antineutrophil cytoplasmic antibody (ANCA)-associated vasculitis. Nephrol Dial Transplant. 2015;30(Suppl 1):i14-22. 10.1093/ndt/gfv022. 25805746 10.1093/ndt/gfv022

[2] Falk RJ, Jennette JC. Anti-neutrophil cytoplasmic autoantibodies with specificity for myeloperoxidase in patients with systemic vasculitis and idiopathic necrotizing and crescentic glomerulonephritis. N Engl J Med. 1988;318(25):1651–7. 10.1056/NEJM198806233182504. 2453802 10.1056/NEJM198806233182504

[3] Jenne DE, Tschopp J, Ludemann J, Utecht B, Gross WL. Wegener’s autoantigen decoded. Nature. 1990;346(6284):520. 10.1038/346520a0. 2377228 10.1038/346520a0

[4] Cornec D, Cornec-Le Gall E, Fervenza FC, Specks U. ANCA-associated vasculitis - clinical utility of using ANCA specificity to classify patients. Nat Rev Rheumatol. 2016;12(10):570–9. 10.1038/nrrheum.2016.123. 27464484 10.1038/nrrheum.2016.123

[5] Stone JH, Merkel PA, Spiera R, Seo P, Langford CA, Hoffman GS, et al. Rituximab versus cyclophosphamide for ANCA-associated vasculitis. N Engl J Med. 2010;363(3):221–32. 10.1056/NEJMoa0909905. 20647199 10.1056/NEJMoa0909905 PMC3137658

[6] Jones RB, Tervaert JW, Hauser T, Luqmani R, Morgan MD, Peh CA, et al. Rituximab versus cyclophosphamide in ANCA-associated renal vasculitis. N Engl J Med. 2010;363(3):211–20. 10.1056/NEJMoa0909169. 20647198 10.1056/NEJMoa0909169

[7] Floege J, Jayne DRW, Sanders J-SF, Tesar V, Rovin BH. KDIGO 2024 Clinical practice guideline for the management of Antineutrophil Cytoplasmic Antibody (ANCA)-associated vasculitis. Kidney Int. 2024;105(3):S71–116. 10.1016/j.kint.2023.10.008. 38388102 10.1016/j.kint.2023.10.008

[8] Geetha D, Hruskova Z, Segelmark M, Hogan J, Morgan MD, Cavero T, et al. Rituximab for treatment of severe renal disease in ANCA associated vasculitis. J Nephrol. 2016;29(2):195–201. 10.1007/s40620-015-0208-y. 25986390 10.1007/s40620-015-0208-y

[9] Casal Moura M, Irazabal MV, Eirin A, Zand L, Sethi S, Borah BJ, et al. Efficacy of rituximab and plasma exchange in antineutrophil cytoplasmic antibody-associated vasculitis with severe kidney disease. J Am Soc Nephrol. 2020;31(11):2688–704. 10.1681/ASN.2019111197. 32826324 10.1681/ASN.2019111197 PMC7608964

[10] Puechal X, Iudici M, Perrodeau E, Bonnotte B, Lifermann F, Le Gallou T, et al. Rituximab vs cyclophosphamide induction therapy for patients with granulomatosis with polyangiitis. JAMA Netw Open. 2022;5(11): e2243799. 10.1001/jamanetworkopen.2022.43799. 36441554 10.1001/jamanetworkopen.2022.43799 PMC9706346

[11] Gulati K, Edwards H, Prendecki M, Cairns TD, Condon M, Galliford J, et al. Combination treatment with rituximab, low-dose cyclophosphamide and plasma exchange for severe antineutrophil cytoplasmic antibody-associated vasculitis. Kidney Int. 2021;100(6):1316–24. 10.1016/j.kint.2021.08.025. 34560140 10.1016/j.kint.2021.08.025

[12] Cortazar FB, Muhsin SA, Pendergraft WF 3rd, Wallace ZS, Dunbar C, Laliberte K, et al. Combination Therapy With Rituximab and Cyclophosphamide for Remission Induction in ANCA Vasculitis. Kidney Int Rep. 2018;3(2):394–402. 10.1016/j.ekir.2017.11.004. 29725643 10.1016/j.ekir.2017.11.004 PMC5932132

[13] Jones RB, Hiemstra TF, Ballarin J, Blockmans DE, Brogan P, Bruchfeld A, et al. Mycophenolate mofetil versus cyclophosphamide for remission induction in ANCA-associated vasculitis: a randomised, non-inferiority trial. Ann Rheum Dis. 2019;78(3):399–405. 10.1136/annrheumdis-2018-214245. 30612116 10.1136/annrheumdis-2018-214245

[14] Reddy V, Dahal LN, Cragg MS, Leandro M. Optimising B-cell depletion in autoimmune disease: is obinutuzumab the answer? Drug Discov Today. 2016;21(8):1330–8. 10.1016/j.drudis.2016.06.009. 27343722 10.1016/j.drudis.2016.06.009

[15] Furie RA, Aroca G, Cascino MD, Garg JP, Rovin BH, Alvarez A, et al. B-cell depletion with obinutuzumab for the treatment of proliferative lupus nephritis: a randomised, double-blind, placebo-controlled trial. Ann Rheum Dis. 2022;81(1):100–7. 10.1136/annrheumdis-2021-220920. 34615636 10.1136/annrheumdis-2021-220920 PMC8762029

[16] Amudala NA, Boukhlal S, Sheridan B, Langford CA, Geara A, Merkel PA, et al. Obinutuzumab as treatment for ANCA-associated vasculitis. Rheumatology (Oxford). 2022;61(9):3814–7. 10.1093/rheumatology/keab916. 34958343 10.1093/rheumatology/keab916

[17] Walsh M, Merkel PA, Peh CA, Szpirt WM, Puechal X, Fujimoto S, et al. Plasma exchange and glucocorticoids in severe ANCA-associated vasculitis. N Engl J Med. 2020;382(7):622–31. 10.1056/NEJMoa1803537. 32053298 10.1056/NEJMoa1803537 PMC7325726

[18] Jayne DRW, Merkel PA, Schall TJ, Bekker P, Group AS. Avacopan for the treatment of ANCA-associated vasculitis. N Engl J Med. 2021;384(7):599–609. 10.1056/NEJMoa2023386. 33596356 10.1056/NEJMoa2023386

[19] Robson J, Doll H, Suppiah R, Flossmann O, Harper L, Hoglund P, et al. Glucocorticoid treatment and damage in the anti-neutrophil cytoplasm antibody-associated vasculitides: long-term data from the European Vasculitis Study Group trials. Rheumatology (Oxford). 2015;54(3):471–81. 10.1093/rheumatology/keu366. 25205825 10.1093/rheumatology/keu366

[20] McGovern D, Williams SP, Parsons K, Farrah TE, Gallacher PJ, Miller-Hodges E, et al. Long-term outcomes in elderly patients with ANCA-associated vasculitis. Rheumatology (Oxford). 2020;59(5):1076–83. 10.1093/rheumatology/kez388. 31794032 10.1093/rheumatology/kez388 PMC7671635

[21] Morris A, Geetha D. PEXIVAS challenges current ANCA-associated vasculitis therapy. Nat Rev Nephrol. 2020;16(7):373–4. 10.1038/s41581-020-0269-6. 32203311 10.1038/s41581-020-0269-6

[22] Pusey CD, Rees AJ, Evans DJ, Peters DK, Lockwood CM. Plasma exchange in focal necrotizing glomerulonephritis without anti-GBM antibodies. Kidney Int. 1991;40(4):757–63. 10.1038/ki.1991.272. 1745027 10.1038/ki.1991.272

[23] Jayne DR, Gaskin G, Rasmussen N, Abramowicz D, Ferrario F, Guillevin L, et al. Randomized trial of plasma exchange or high-dosage methylprednisolone as adjunctive therapy for severe renal vasculitis. J Am Soc Nephrol. 2007;18(7):2180–8. 10.1681/ASN.2007010090. 17582159 10.1681/ASN.2007010090

[24] Walsh M, Collister D, Zeng L, Merkel PA, Pusey CD, Guyatt G, et al. The effects of plasma exchange in patients with ANCA-associated vasculitis: an updated systematic review and meta-analysis. BMJ. 2022;376: e064604. 10.1136/bmj-2021-064604. 35217545 10.1136/bmj-2021-064604

[25] Hellmich B, Sanchez-Alamo B, Schirmer JH, Berti A, Blockmans D, Cid MC, et al. EULAR recommendations for the management of ANCA-associated vasculitis: 2022 update. Ann Rheum Dis. 2023. 10.1136/ard-2022-223764. 10.1136/ard-2022-223764 36927642

[26] Uto K, Yanagi S, Tsubouchi H, Matsumoto N, Nakazato M. Successful treatment of steroid-refractory double-positive ANCA and anti-GBM disease with a combination of plasma exchange and immunosuppression: a case report and literature review. Respir Med Case Rep. 2018;25:242–6. 10.1016/j.rmcr.2018.09.016. 30302307 10.1016/j.rmcr.2018.09.016 PMC6174835

[27] McAdoo SP, Tanna A, Hruskova Z, Holm L, Weiner M, Arulkumaran N, et al. Patients double-seropositive for ANCA and anti-GBM antibodies have varied renal survival, frequency of relapse, and outcomes compared to single-seropositive patients. Kidney Int. 2017;92(3):693–702. 10.1016/j.kint.2017.03.014. 28506760 10.1016/j.kint.2017.03.014 PMC5567410

[28] Klemmer PJ, Chalermskulrat W, Reif MS, Hogan SL, Henke DC, Falk RJ. Plasmapheresis therapy for diffuse alveolar hemorrhage in patients with small-vessel vasculitis. Am J Kidney Dis. 2003;42(6):1149–53. 10.1053/j.ajkd.2003.08.015. 14655185 10.1053/j.ajkd.2003.08.015

[29] Pagnoux C, Mahr A, Hamidou MA, Boffa JJ, Ruivard M, Ducroix JP, et al. Azathioprine or methotrexate maintenance for ANCA-associated vasculitis. N Engl J Med. 2008;359(26):2790–803. 10.1056/NEJMoa0802311. 19109574 10.1056/NEJMoa0802311

[30] Silva F, Specks U, Kalra S, Hogan MC, Leung N, Sethi S, et al. Mycophenolate mofetil for induction and maintenance of remission in microscopic polyangiitis with mild to moderate renal involvement–a prospective, open-label pilot trial. Clin J Am Soc Nephrol. 2010;5(3):445–53. 10.2215/CJN.06010809. 20093349 10.2215/CJN.06010809 PMC2827580

[31] Hiemstra TF, Walsh M, Mahr A, Savage CO, de Groot K, Harper L, et al. Mycophenolate mofetil vs azathioprine for remission maintenance in antineutrophil cytoplasmic antibody-associated vasculitis: a randomized controlled trial. JAMA. 2010;304(21):2381–8. 10.1001/jama.2010.1658. 21060104 10.1001/jama.2010.1658

[32] Guillevin L, Pagnoux C, Karras A, Khouatra C, Aumaitre O, Cohen P, et al. Rituximab versus azathioprine for maintenance in ANCA-associated vasculitis. N Engl J Med. 2014;371(19):1771–80. 10.1056/NEJMoa1404231. 25372085 10.1056/NEJMoa1404231

[33] Smith RM, Jones RB, Specks U, Bond S, Nodale M, Al-Jayyousi R, et al. Rituximab versus azathioprine for maintenance of remission for patients with ANCA-associated vasculitis and relapsing disease: an international randomised controlled trial. Ann Rheum Dis. 2023;82(7):937–44. 10.1136/ard-2022-223559. 36958796 10.1136/ard-2022-223559 PMC10313987

[34] Charles P, Terrier B, Perrodeau E, Cohen P, Faguer S, Huart A, et al. Comparison of individually tailored versus fixed-schedule rituximab regimen to maintain ANCA-associated vasculitis remission: results of a multicentre, randomised controlled, phase III trial (MAINRITSAN2). Ann Rheum Dis. 2018;77(8):1143–9. 10.1136/annrheumdis-2017-212878. 29695500 10.1136/annrheumdis-2017-212878

[35] Jayne D, Blockmans D, Luqmani R, Moiseev S, Ji B, Green Y, et al. Efficacy and safety of belimumab and azathioprine for maintenance of remission in antineutrophil cytoplasmic antibody-associated vasculitis: a randomized controlled study. Arthritis Rheumatol. 2019;71(6):952–63. 10.1002/art.40802. 30666823 10.1002/art.40802 PMC6593987

[36] Karras A, Pagnoux C, Haubitz M, Groot K, Puechal X, Tervaert JWC, et al. Randomised controlled trial of prolonged treatment in the remission phase of ANCA-associated vasculitis. Ann Rheum Dis. 2017;76(10):1662–8. 10.1136/annrheumdis-2017-211123. 28546260 10.1136/annrheumdis-2017-211123

[37] Charles P, Perrodeau E, Samson M, Bonnotte B, Neel A, Agard C, et al. Long-term rituximab use to maintain remission of antineutrophil cytoplasmic antibody-associated vasculitis: a randomized trial. Ann Intern Med. 2020;173(3):179–87. 10.7326/M19-3827. 32479166 10.7326/M19-3827

[38] Delestre F, Charles P, Karras A, Pagnoux C, Neel A, Cohen P, et al. Rituximab as maintenance therapy for ANCA-associated vasculitides: pooled analysis and long-term outcome of 277 patients included in the MAINRITSAN trials. Ann Rheum Dis. 2024;83(2):233–41. 10.1136/ard-2023-224623. 37918894 10.1136/ard-2023-224623

[39] Chung SA, Langford CA, Maz M, Abril A, Gorelik M, Guyatt G, et al. 2021 American College of Rheumatology/Vasculitis Foundation Guideline for the Management of Antineutrophil Cytoplasmic Antibody-Associated Vasculitis. Arthritis Rheumatol. 2021;73(8):1366–83. 10.1002/art.41773. 34235894 10.1002/art.41773 PMC12327957

[40] Haas M, Eustace JA. Immune complex deposits in ANCA-associated crescentic glomerulonephritis: a study of 126 cases. Kidney Int. 2004;65(6):2145–52. 10.1111/j.1523-1755.2004.00632.x. 15149327 10.1111/j.1523-1755.2004.00632.x

[41] Chen M, Xing GQ, Yu F, Liu G, Zhao MH. Complement deposition in renal histopathology of patients with ANCA-associated pauci-immune glomerulonephritis. Nephrol Dial Transplant. 2009;24(4):1247–52. 10.1093/ndt/gfn586. 18940884 10.1093/ndt/gfn586

[42] Augusto JF, Langs V, Demiselle J, Lavigne C, Brilland B, Duveau A, et al. Low serum complement C3 levels at diagnosis of renal ANCA-associated vasculitis is associated with poor prognosis. PLoS ONE. 2016;11(7): e0158871. 10.1371/journal.pone.0158871. 27391243 10.1371/journal.pone.0158871 PMC4938207

[43] Xiao H, Schreiber A, Heeringa P, Falk RJ, Jennette JC. Alternative complement pathway in the pathogenesis of disease mediated by anti-neutrophil cytoplasmic autoantibodies. Am J Pathol. 2007;170(1):52–64. 10.2353/ajpath.2007.060573. 17200182 10.2353/ajpath.2007.060573 PMC1762697

[44] Xiao H, Dairaghi DJ, Powers JP, Ertl LS, Baumgart T, Wang Y, et al. C5a receptor (CD88) blockade protects against MPO-ANCA GN. J Am Soc Nephrol. 2014;25(2):225–31. 10.1681/ASN.2013020143. 24179165 10.1681/ASN.2013020143 PMC3904560

[45] Jayne DRW, Bruchfeld AN, Harper L, Schaier M, Venning MC, Hamilton P, et al. Randomized trial of C5a receptor inhibitor avacopan in ANCA-associated vasculitis. J Am Soc Nephrol. 2017;28(9):2756–67. 10.1681/ASN.2016111179. 28400446 10.1681/ASN.2016111179 PMC5576933

[46] Merkel PA, Niles J, Jimenez R, Spiera RF, Rovin BH, Bomback A, et al. Adjunctive treatment with avacopan, an oral c5a receptor inhibitor, in patients with antineutrophil cytoplasmic antibody-associated vasculitis. ACR Open Rheumatol. 2020;2(11):662–71. 10.1002/acr2.11185. 33128347 10.1002/acr2.11185 PMC7672305

[47] Manenti L, Urban ML, Maritati F, Galetti M, Vaglio A. Complement blockade in ANCA-associated vasculitis: an index case, current concepts and future perspectives. Intern Emerg Med. 2017;12(6):727–31. 10.1007/s11739-017-1636-6. 28191609 10.1007/s11739-017-1636-6

[48] Ribes D, Belliere J, Piedrafita A, Faguer S. Glucocorticoid-free induction regimen in severe ANCA-associated vasculitis using a combination of rituximab and eculizumab. Rheumatology (Oxford). 2019;58(12):2335–7. 10.1093/rheumatology/kez190. 31102526 10.1093/rheumatology/kez190

[49] Kitamura F, Yamaguchi M, Nishimura M, Katsuno T, Ito M, Sugiyama H, et al. Anti-neutrophil cytoplasmic antibody-associated vasculitis complicated by thrombotic microangiopathy with posterior reversible encephalopathy syndrome successfully treated with eculizumab: a case report. Mod Rheumatol Case Rep. 2022;6(2):254–9. 10.1093/mrcr/rxac029. 35425980 10.1093/mrcr/rxac029

[50] Merkel P HB, Pfaff A, Müller C, Startseva E, Jayne D. A randomized, double-blind, phase II study of glucocorticoid replacement by vilobelimab, an anti-C5a monoclonal antibody, in ANCA-associated vasculitis [abstract]. Arthritis Rheumatol. 2022;74(suppl 9).

